# On DNA numerical representations for genomic similarity computation

**DOI:** 10.1371/journal.pone.0173288

**Published:** 2017-03-21

**Authors:** Gerardo Mendizabal-Ruiz, Israel Román-Godínez, Sulema Torres-Ramos, Ricardo A. Salido-Ruiz, J. Alejandro Morales

**Affiliations:** Departamento de Ciencias Computacionales, División de Electrónica y Computación, Universidad de Guadalajara, Guadalajara, Jalisco, México; Centre for Research and Technology-Hellas, GREECE

## Abstract

Genomic signal processing (GSP) refers to the use of signal processing for the analysis of genomic data. GSP methods require the transformation or mapping of the genomic data to a numeric representation. To date, several DNA numeric representations (DNR) have been proposed; however, it is not clear what the properties of each DNR are and how the selection of one will affect the results when using a signal processing technique to analyze them. In this paper, we present an experimental study of the characteristics of nine of the most frequently-used DNR. The objective of this paper is to evaluate the behavior of each representation when used to measure the similarity of a given pair of DNA sequences.

## Introduction

Genomic signal processing (GSP) refers to the use of signal processing theory, algorithms, and mathematical methods for the analysis, transformation, and interpretation of the information contained in genomic data. It has been an active field of research for the past 25 years. While most current GSP methods focus on identifying protein-coding regions in DNA sequences (e.g., [1–10]), other applications include searching for genomic repeats [11], determining the structural, thermodynamic, and bending properties of DNA [12], biological sequence querying [13], estimating of DNA sequence similarity [14–16], and sequence alignment [17].

GSP methods require the transformation or mapping of the genomic information usually represented as a string of characters (i.e., A, T, G and C) to a numeric representation in the form of a single or multidimensional array of numeric values (i.e., a signal) [18]. Current DNA numerical representations (DNR) may be divided into three categories: single-value mapping, multidimensional sequence mapping, and cumulative sequence mapping.

Single-value representations are characterized by the use of a single one-dimensional numerical value for each nucleotide in the DNA sequence. In this category we find: (i) “integer representation”, where a numeric vector is generated by replacing each of the four possible letters of the nucleotide by a fixed integer value [19]; (ii) “real number representation”, which employs positive decimal values for the pyrimidines (i.e., **A** and **G**), and negative decimal values for the purines (**C** and **T**) [20, 21]; (iii) “paired numeric representation”, which incorporates the complementarity property of the nucleotides in the DNA strain [5]; (iv) “atomic number representation”, which assigns the atomic number of each nucleotide [22]; and, (v) “electron-ion” interaction potential representation (EIIP), which employs numeric values that represent the distribution of the free electron’s energies along the DNA sequence [23].

Multidimensional representations replace every nucleotide in the DNA sequence with a vector that represents a point in a space of two or more dimensions. In this category, we find: (i) “Voss representation”, which employs four binary indicator sequences to denote the presence of a nucleotide of each type [24]; and (ii) “Tetrahedron representation”, in which each nucleotide corresponds to a vertex of a three-dimensional structure that is characterized by having equal distances between every pair of vortices [25].

Cumulative representations can use single or multidimensional vectors, and are characterized by employing a random walk model in which a curve is constructed by the aggregate contribution of consecutive numeric values assigned to each nucleotide. In this category we find (i) “DNA walk representation” consists in taking a step upwards if the nucleotide is a pyrimidine, and downwards if it is a purine [26]; and (ii) “Z-curve representation”, which constructs a three-dimensional curve in which the first dimension relates to the distributions of the types of nitrogenous base rings (purines vs. pyrimidines), while the second reflects the type of chemical functional groups (i.e., amino vs. keto), and the third represents the strength of the hydrogen bonds in the nucleotide molecules (i.e., strong H bonds vs. weak H-bonds) [27].

To date, no DNR can be considered the “gold standard” nor is there any study or comparison of the properties of the different DNRs in a common task. In this paper, we present an experimental study and comparison of the characteristics of nine common DNRs when used to estimate the similarity between DNA sequences, employing the frequency power spectrum obtained by the fast Fourier transform (FFT). The principle contribution of this paper is its the exploration of the characteristics of the existing DNRs, which helps to provide insight into the features that may be desirable for proposing new DNRs and GSP methods.

## Materials and methods

Nine of the DNRs in the literature were selected for analysis and comparison (Table 1). For each DNR, we performed synthetic and biological data experiments consisting of the computation of pairwise DNA sequence similarity. The details of the proposed experimental methodology are described in the following section.

**Table 1:** Selected DNA numerical representations.

	Name	Numeric representation	Example for sequence X = [*AACTGT*]
1	Integer	$\hat{X}\left(i\right)=\{\begin{array}{cc} 3 & \text{if}\;X(i)=\text{G}\\ 2 & \text{if}\;X(i)=\text{A}\\ 1 & \text{if}\;X(i)=\text{C}\\ 0 & \text{if}\;X(i)=\text{T} \end{array}$	$\hat{X}=[2,2,1,0,3,0]$
2	Real	$\hat{X}\left(i\right)=\{\begin{array}{cc} -0.5 & \text{if}\;X(i)=\text{G}\\ -1.5 & \text{if}\;X(i)=\text{A}\\ 0.5 & \text{if}\;X(i)=\text{C}\\ 1.5 & \text{if}\;X(i)=\text{T} \end{array}$	$\hat{X}=[-1.5,-1.5,0.5,1.5,-0.5,1.5]$
3	EIIP	$\hat{X}\left(i\right)=\{\begin{array}{cc} 0.0806 & \text{if}\;X(i)=\text{G}\\ 0.1260 & \text{if}\;X(i)=\text{A}\\ 0.1340 & \text{if}\;X(i)=\text{C}\\ 0.1335 & \text{if}\;X(i)=\text{T} \end{array}$	$\hat{X}=[0.1260,0.1260,0.1340,0.1335,0.0806,0.1335]$
4	Atomic Number	$\hat{X}\left(i\right)=\{\begin{array}{cc} 78 & \text{if}\;X(i)=\text{G}\\ 70 & \text{if}\;X(i)=\text{A}\\ 58 & \text{if}\;X(i)=\text{C}\\ 66 & \text{if}\;X(i)=\text{T} \end{array}$	$\hat{X}=[70,70,58,66,78,66]$
5	Paired Numeric	$\begin{array}{c} \hat{X}\left(i\right)=\{\begin{array}{cc} 1 & \text{if}\;X(i)=A\vee T\\ -1 & \text{otherwise} \end{array} \end{array}$	$\begin{array}{c} \hat{X}=[1,1,-1,1,-1,1] \end{array}$
6	Voss	$\begin{array}{c} {\hat{X}}_{1}\left(i\right)=\{\begin{array}{cc} 1 & \text{if}\;X(i)=A\\ 0 & \text{otherwise} \end{array}\\ {\hat{X}}_{2}\left(i\right)=\{\begin{array}{cc} 1 & \text{if}\;X(i)=G\\ 0 & \text{otherwise} \end{array}\\ {\hat{X}}_{3}\left(i\right)=\{\begin{array}{cc} 1 & \text{if}\;X(i)=C\\ 0 & \text{otherwise} \end{array}\\ {\hat{X}}_{4}\left(i\right)=\{\begin{array}{cc} 1 & \text{if}\;X(i)=T\\ 0 & \text{otherwise} \end{array} \end{array}$	$\begin{array}{c} {\hat{X}}_{1}=[1,1,0,0,0,0]\\ {\hat{X}}_{2}=[0,0,0,0,1,0]\\ {\hat{X}}_{3}=[0,0,1,0,0,0]\\ {\hat{X}}_{4}=[0,0,0,1,0,1] \end{array}$
7	Tetrahedron	$\begin{array}{c} {\hat{X}}_{1}\left(i\right)=\{\begin{array}{cc} \frac{2\sqrt{2}}{3} & \text{if}\;X(i)=T\\ -\frac{\sqrt{2}}{3} & \text{if}\;X(i)=C\vee G\\ 0 & \text{otherwise} \end{array}\\ {\hat{X}}_{2}\left(i\right)=\{\begin{array}{cc} \frac{\sqrt{6}}{3} & \text{if}\;X(i)=C\\ -\frac{\sqrt{6}}{3} & \text{if}\;X(i)=G\\ 0 & \text{otherwise} \end{array}\\ {\hat{X}}_{3}\left(i\right)=\{\begin{array}{cc} 1 & \text{if}\;X(i)=A\\ -\frac{1}{3} & \text{otherwise} \end{array} \end{array}$	$\begin{array}{c} {\hat{X}}_{1}=[0,0,-\frac{\sqrt{2}}{3},\frac{2\sqrt{2}}{3},-\frac{\sqrt{2}}{3},2\frac{1}{3}]\\ {\hat{X}}_{2}=[0,0,\frac{\sqrt{6}}{3},0,-\frac{\sqrt{6}}{3},0]\\ {\hat{X}}_{3}=[1,1,-\frac{1}{3},-\frac{1}{3},-\frac{1}{3},-\frac{1}{3}] \end{array}$
8	Z-Curve	$\begin{array}{c} {\hat{X}}_{1}\left(i\right)=\{\begin{array}{cc} X(i-1)+1 & \text{if}\;X(i)=T\vee G\\ X(i-1)+(-1) & \text{otherwise} \end{array}\\ {\hat{X}}_{2}\left(i\right)=\{\begin{array}{cc} X(i-1)+1 & \text{if}\;X(i)=A\vee C\\ X(i-1)+(-1) & \text{otherwise} \end{array}\\ {\hat{X}}_{3}\left(i\right)=\{\begin{array}{cc} X(i-1)+1 & \text{if}\;X(i)=A\vee T\\ X(i-1)+(-1) & \text{otherwise} \end{array} \end{array}$	$\begin{array}{c} {\hat{X}}_{1}=[-1,-2,-3,-2,-1,0]\\ {\hat{X}}_{2}=[1,2,3,2,1,0]\\ {\hat{X}}_{3}=[1,2,1,2,1,2] \end{array}$
9	DNA walk	$\begin{array}{c} \hat{X}\left(i\right)=\{\begin{array}{cc} X(i-1)+1 & \text{if}\;X(i)=C\vee T\\ X(i-1)+(-1) & \text{otherwise} \end{array} \end{array}$	$\begin{array}{c} \hat{X}=[-1,-2,-1,0,-1,0] \end{array}$

### Sequence similarity computation

Consider a DNA sequence *α* (e.g., *α* = *ATTCGCAT*…) and let ${\hat{X}}^{\alpha }$ denote the digital signal version of that sequence that has been obtained using a DNR method. By applying the FFT to ${\hat{X}}^{\alpha }$ it is possible to compute its power spectral density (PSD) ${\hat{S}}^{\alpha }$, which describes how the power of the signal (energy per unit time) is distributed over the different frequencies [28].

Consider two DNA signals ${\hat{X}}^{\alpha }$ and ${\hat{X}}^{\beta }$ corresponding to two DNA sequences *α* and *β*, respectively. The relatedness or similarity score of these two sequences can be estimated by comparing their frequency power spectra $d({\hat{S}}^{\alpha },{\hat{S}}^{\beta })$ using a similarity metric.

In this work, we explore four widely-used metrics: Euclidean distance [29], Normalized Squared Euclidean distance [30], Correlation coefficient [29], and Manhattan distance [29]. To compare the PSD of two signals, both spectra must have the same number of elements *k*, and every element in both vectors must correspond to the same frequency component. However, since the length of the signal representation of two different DNA sequences can differ, this condition may not be satisfied. To overcome this challenge, we apply a zero padding to the DNA signal with the smaller length before computing the FFT [31]. Also, the first entry of a power spectrum (e.g., ${\hat{S}}^{\alpha }\left(0\right)$) is known as the zero-frequency (DC) component and represents the average intensity of the DNA signal. In this work, we chose not to consider the DC component in the spectrum comparisons for two reasons: (i) this value does not provide information about the possible patterns present in the DNA sequences; and, (ii) this value is affected by the zero padding, which will have an impact on the computed similarity score.

#### Euclidean and normalized squared euclidean distances

The Euclidean distance Eq (1) is a metric used to define the distance between two points in an *N*-dimensional space. By considering each *k* frequency component of a DNA signal spectra as a dimension, a DNA sequence may be represented as a point in a *k*-dimensional space. Therefore, the Euclidean distance can be employed to determine the relatedness or similarity between sequences. A Euclidean distance of zero can be interpreted as meaning that the two DNA sequences are identical or closely-related, while a larger value means that the sequences are different. Additionally, since the Euclidean distance is unbounded (i.e., there is no limit for the largest value), we compute the normalized squared Euclidean distance (Eq (2)), which provides similarity values in the interval [01].

$$\begin{array}{c} d_{E}\left({\hat{S}}^{\alpha },{\hat{S}}^{\beta }\right)=\sqrt[{2}]{\sum_{f=1}^{k}\;{\left({\hat{S}}^{\alpha }\left(f\right)-{\hat{S}}^{\beta }\left(f\right)\right)}^{2}}, \end{array}$$ (1)

$$\begin{array}{c} d_{NSE}\left({\hat{S}}^{\alpha },{\hat{S}}^{\beta }\right)=\frac{1}{2}*\frac{{∥\left({\hat{S}}^{\alpha }-\bar{{\hat{S}}^{\alpha }}\right)-\left({\hat{S}}^{\beta }-\bar{{\hat{S}}^{\beta }}\right)∥}^{2}}{{∥\left({\hat{S}}^{\alpha }-\bar{{\hat{S}}^{\alpha }}\right)∥}^{2}+{∥\left({\hat{S}}^{\beta }-\bar{{\hat{S}}^{\beta }}\right)∥}^{2}} \end{array}$$ (2)

#### Manhattan distance

The Manhattan distance described in Eq (3) (also known as Taxicab geometry or L1-Norm), is also used to determine the distance between two points in an *N*-dimensional space; however, it considers distance only in orthogonal directions. This metric is usually used to assess the differences in discrete space distributions, in contrast to the Euclidean metric. Thus, this property makes it suitable for use as a measure of similarity between the PSD of DNA signals.

$$\begin{array}{c} d_{l1}\left({\hat{S}}^{\alpha },{\hat{S}}^{\beta }\right)={∥{\hat{S}}^{\alpha }-{\hat{S}}^{\beta }∥}_{1}=\sum_{i=1}^{n}\;\left|{\hat{S}}_{i}^{\alpha }-{\hat{S}}_{i}^{\beta }\right| \end{array}$$ (3)

#### Correlation coefficient

The correlation coefficient (Eq (4)) measures the strength and direction of a linear relationship between two variables and so can be used to measure the degree of similarity between the PSD of two DNA signals. The correlation coefficient is bounded in the interval [01]. In general, a correlation value greater than 0.8 is generally assumed as strong, whereas a correlation smaller than 0.5 is generally assumed as weak.

$$\begin{array}{c} corr\left({\hat{S}}^{\alpha },{\hat{S}}^{\beta }\right)=\frac{\sum_{f=1}^{k}\left({\hat{S}}^{\alpha }\left(f\right)-\bar{{\hat{S}}^{\alpha }}\right)\left({\hat{S}}^{\beta }\left(f\right)-\bar{{\hat{S}}^{\beta }}\right)}{\sqrt{\sum_{f=1}^{k}{\left({\hat{S}}^{\alpha }\left(f\right)-\bar{{\hat{S}}^{\alpha }}\right)}^{2}\sum_{f=2}^{k}{\left({\hat{S}}^{\beta }\left(f\right)-\bar{{\hat{S}}^{\beta }}\right)}^{2}}} \end{array}$$ (4)

where

$$\begin{array}{c} \bar{\hat{S}}=\frac{1}{k}\;\sum_{f=1}^{k}\;\hat{S}\left(f\right). \end{array}$$ (5)

### Synthetic data experiments

#### Data generation

To evaluate how different changes in a DNA sequence will affect the similarity score when using a DNR, we generated a baseline DNA sequence of length 1,000 where each element was selected randomly with an equal probability of 0.25 for each type of nucleotide (i.e., A, C, G, T). A total of 42 datasets were generated that corresponded to the combinations of seven types of modifications (i.e., the three basic types of changes: insertion (i), deletion (d), substitution (s); and their combinations: insertion and deletion (i-d), insertion and substitution (i-s), deletion and substitution (d-s), insertion, deletion, and substitution (i-d-s)), and six percentages of change (i.e., 1%, 2%, 4%, 8%, 16% and 32%) with respect to the baseline sequence. For each type of change, the position of the nucleotide to be inserted, removed, or replaced was selected randomly using uniform distribution. The kind of nucleotide to be inserted or replaced was also selected using an equal probability of 0.25 for each type of nucleotide. For each of the 42 datasets, we generated a sample of 400 sequences out of the total number of possible variations of the baseline sequence. The sample size of 400 was determined by computing the minimal number of modified sequences needed for statistically significant experiments with a confidence interval of *Z* = 1.96, an expected true proportion of *p* = 0.5, and a confidence interval of *c* = 0.05 [32].

We performed three experiments using the synthetic data:

1. The first experiment was designed to evaluate how a DNR is affected by the different types of change. We computed the mean similarity score of all the modified sequences within every data set compared to the baseline sequence using the Euclidean distance, normalized squared Euclidean distance, Manhattan distance, and correlation coefficient.
2. The second experiment was designed to evaluate how the different percentages of change affect the frequency components of the power spectrum generated with a DNR. To this end, we computed the variance of the similarity score of all the modified sequences within the i-d-s data set, then we divided the power spectrum frequency axis into ten frequency ranges. For each range of frequencies, we computed the average variance and mapped it to a color value to generate an image that depicts the changes in the variance of the frequency components with respect to the percentage of change.
3. The third experiment consisted in evaluating the genetic similarity score obtained with each selected DNR, when comparing a DNA sequence with its corresponding complementary sequence (e.g., the complementary sequence of ATCG is TAGC), and its reverse complementary sequence (e.g., the reverse complementary sequence of ATCG is CGAT). To achieve this, we generated the complementary sequence of the baseline sequence, and computed the similarity by comparing the power spectra with the Euclidean distance, normalized squared Euclidean distance, Manhattan distance, and correlation coefficient.

### Biological data experiments

**Fig 1:**
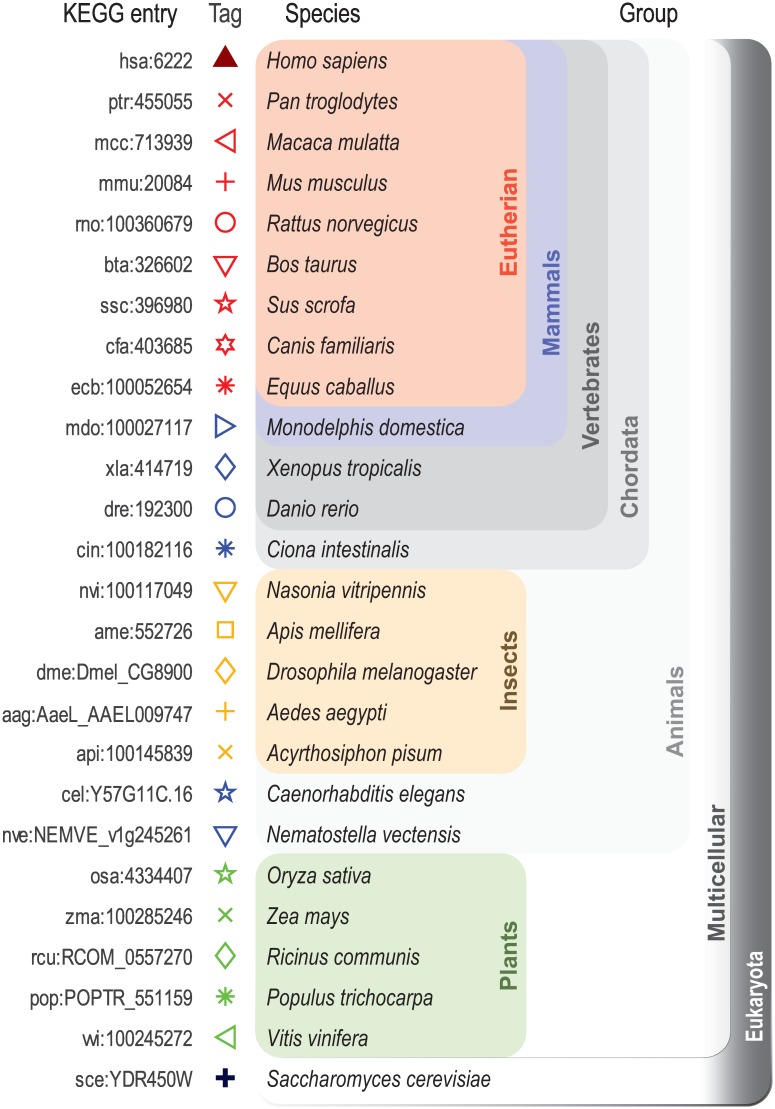
Biological species selected for gene RP-S18 similarity comparison.

To evaluate the characteristics of the selected DNRs for estimating the similarity between real biological sequences, we generated a database consisting of the DNA sequences that correspond to the ribosomal protein encoding gene RP-S18 [33], downloaded from the Kyoto Encyclopedia of Genes and Genomes (KEGG) database [34, 35]. The main reason for employing the RP-S18 is that this gene can be found in all eukaryotes. Thus, each sequence represents one species in the eukaryote tree, allowing us to evaluate the performance of each DNR in computing the similarity between highly-related species (e.g., *H. sapiens* vs. *P. troglodytes*), as well as distantly-related species (e.g., *H. sapiens* vs. *S. cerevisiae*). Twenty-six sequences were selected in order to generate various clusters that were highly-distinct from each other, i.e., eutherians, insects, and plants. Furthermore, at least one sequence was located outside every group (e.g., *M. domestica* is external to eutherians and together they constitute mammals, for which there are two sequences external to them, and so on), where *S. Cerevisiae* is the furthest external sequence. Fig 1 depicts the species selected for the RP-S18 gene, organized according to the taxonomy tree.

Experiments that consisted in computing the pairwise similarity score of every DNA sequence compared to (i) *H. sapiens* (representing the mammals group), and (ii) *P. Saccharomyces* (representing the species external to all others) were performed employing the Euclidean distance, normalized squared Euclidean distance, Manhattan distance, and correlation coefficient.

In the first experiment, the expectation was that the species belonging to the mammals group (red) would be clustered together with a high similarity score compared to *H. sapiens*, and that the insects (orange), and plants (green) species would be grouped with their corresponding groups with a lower similarity score with respect to all eutherians. On the other hand, it is expected that the most external species, *S. Cerevisiae*, would obtain the lowest similarity score with respect to *H. sapiens*. In the second experiment, the expectation was that every species would obtain low similarity scores with respect to *S. cerevisisae*, with no a particular grouping order.

Additionally, we performed a second set of experiments using the Cytochome C oxidase subunit 1 (COX1), a widely-known gene that has been branded as a general molecular marker [36]. A total of 41 sequences were obtained from the KEGG database (Orthology: K02256), corresponding to 17 mammals, 6 insects, 7 plants, 9 other vertebrates that can be located between the mammals and the insects, 1 organism located between the insects and plants, and the yeast *S. cerevisiae* as the external Eukaryota group. We performed comparisons of each group with respect to one organism: *H. sapiens* for mammals, *D. melanogaster* for insects, *O. sativa* for plants, and the *S. cerevisiae* as the external group.

We selected the COX1 gene because of its ability to allow the differentiation from Phyla to Order with a mean pairwise divergence value of 11.3% among animals [37]. While it can dissect the insect order appropriately [37] and perform reasonably well for all vertebrates [38, 39], its value has been questioned for plants, where its mutation rate is even slower and chloroplast genes are preferred [40].

Based on this rationale, the expectation was that in our experiment that compared sequences with respect to mammals or insects, the animals would distribute adequately. Also, when comparing with respect to plants, two groups, that of plants and those of the rest with seemingly undifferentiated clumps, would appear clearly.

**Fig 2:**
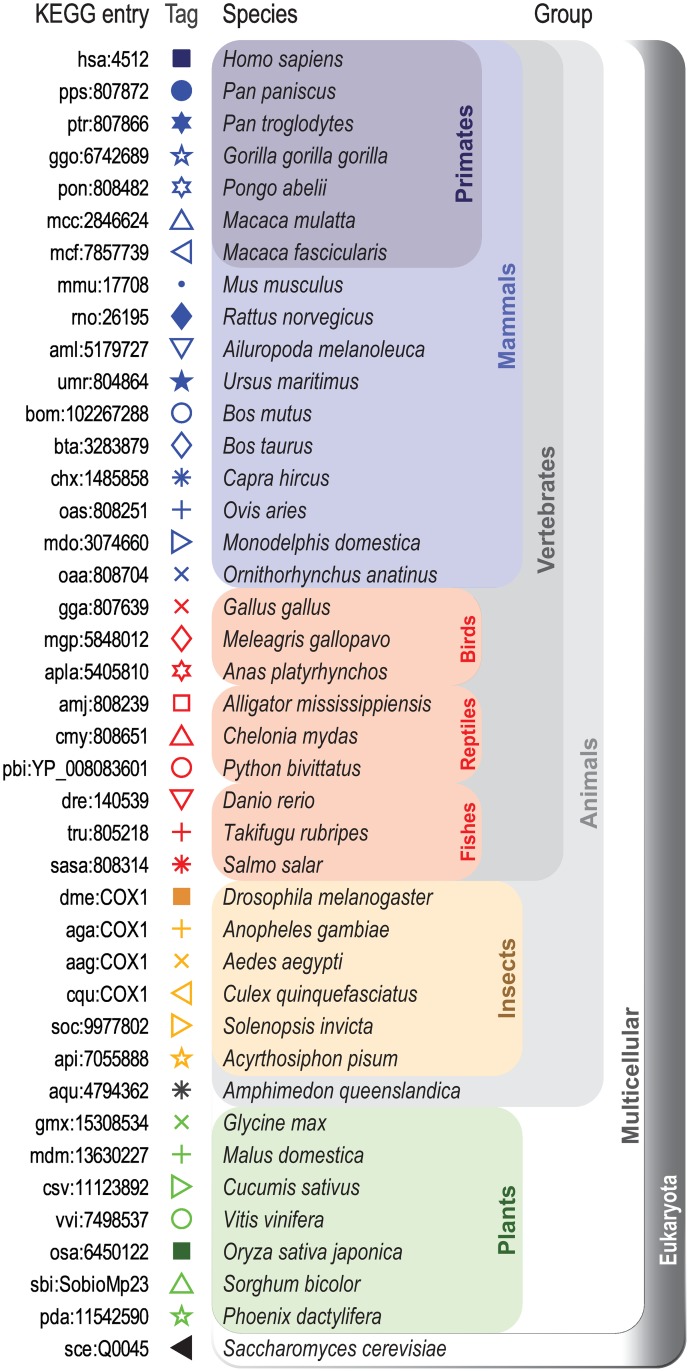
Biological species selected for gene COX1 similarity comparison.

Fig 2 depicts the selected species for the COX1 genes organized according to the taxonomy tree.

## Results

### Synthetic data results

**Fig 3:**
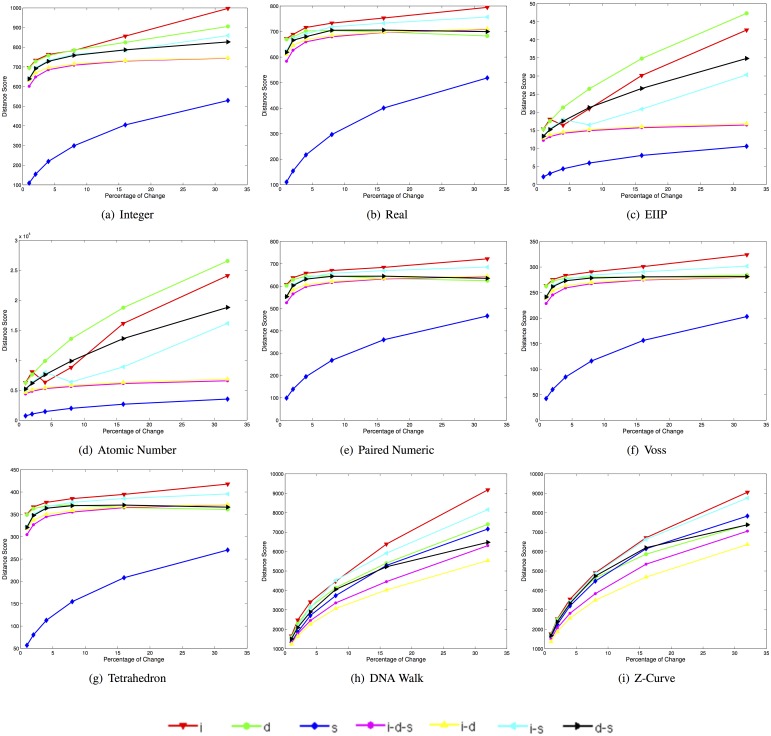
Mean Euclidean distance scores for 400 synthetic sequences in each one of the 42 datasets when using each of the selected DNR (i: insertion, d: deletion, s: substitution, i-d-s: insertion-deletion-substitution, i-d: insertion-deletion, i-s: insertion-substitution, d-s: deletion-substitution).

Fig 3 depicts plots of the mean Euclidean distance scores for 400 synthetic sequences in each one of the 42 datasets when using the selected DNRs.

Note that the increases in the mean Euclidean distance score for the real, paired numeric, Voss, and tetrahedron representations present a similar behavior for all types of changes despite the fact that they correspond to different DNR types (see Section 1). For these DNRs, the curve with the shortest distance scores corresponds to substitutions. The curves corresponding to deletions-substitutions, insertions-deletions, and insertions-deletions-substitutions present notable differences when a small percentage of changes are present, and almost an identical rate of increase after changes above 16%.

The curve corresponding to deletions exhibits a decrease in the Euclidean distance score for changes above 4% for the real, paired numeric, and tetrahedron representations. Finally, the curves corresponding to insertion-substitutions and insertions are the most distant from the baseline sequence in these DNRs. For the integer representation, the curve with the smallest distance with respect to the baseline sequence is also the one corresponding to substitutions.

Note that there is an important difference between the distance scores for the remaining types of changes, with the exception of the curves corresponding to insertions-deletions and insertion-deletion-substitutions the rate of change is very similar. The plots corresponding to the EEIP and atomic number representations are also almost identical, with slight differences in the rate of increase of distance for the curve corresponding to insertions.

Note as well that there is a brief decrease in the distance score at 4% for insertions, and at 8% for the insertion-substitution curves. It is notable that for the DNRs of cumulative type (i.e., DNA walk and Z-curve), the curve corresponding to substitutions is not the one with the lowest Euclidean distance score, as is the case in the other DNRs. For these DNRs, the lowest distance scores correspond to the insertion-deletions, followed by the insertion-deletions-substitutions. Finally, note that the deletion, substitution, and insertion-deletion curves present notable differences in their order in both plots after changes by 16%.

Table 2 lists the angle (in degrees) of the rate of change in the mean Euclidean distance scores for the type of change corresponding to insertions-deletions-substitutions, for five ranges of the percentage of changes.

**Table 2:** Angle (in degrees) of the rate of change in the mean Euclidean distance scores for the type of change corresponding to insertions-deletions-substitutions, for five ranges of the percentage of changes (2-1, 4-2, 8-4, 16-8, 32-16).

	Angle of the Rate of Change					Score by Percentage of Change					
DNR	2-1	4-2	8-4	16-8	32-16	1	2	4	8	16	32
Integer	88.8	86.9	80.2	69.3	42.7	601.37	648.64	685.70	708.74	729.91	744.66
Real	88.7	86.5	78.5	65.0	37.2	583.68	626.69	659.76	679.33	696.50	708.65
EIIP	45.6	25.0	10.1	5.9	2.6	12.22	13.24	14.18	14.89	15.71	16.45
Atomic Number	89.9	89.7	89.3	89.1	88.0	4367.13	4812.52	5258.03	5605.61	6105.16	6568.63
Paired Numeric	88.5	86.5	77.8	63.8	34.6	525.94	565.33	597.74	616.22	632.44	643.48
DNA Walk	89.9	89.8	89.7	89.6	89.5	1309.21	1772.64	2451.74	3360.19	4453.91	6306.96
Voss	86.6	81.7	64.1	42.5	18.4	228.48	245.30	259.07	267.32	274.65	279.95
Tetrahedron	87.4	83.7	68.8	51.0	23.0	304.65	326.77	344.82	355.11	364.98	371.77
Z-Curve	89.9	89.8	89.8	89.7	89.5	1500.43	2066.82	2826.21	3836.01	5351.83	7053.40

Note that for almost all DNRs the angles are close to 90° in the range of 1%–2%, which implies that small differences between two DNA sequences will produce high Euclidean distance scores. The exception to this is the EIIP representation, for which the angle is close to 45°. This DNR presents smaller angles than the others, which means that the Euclidean distance score will not be dramatically affected even when there are great differences between a pair of DNA sequences. The integer and real representations behave similarly in terms of angles, as do as the Voss and tetrahedron representations. The angles corresponding to the atomic number, Z-curve, and DNA walk representations are large for all the ranges, which indicates that any magnitude of difference between two DNA sequences will produce large Euclidean distance scores.

**Fig 4:**
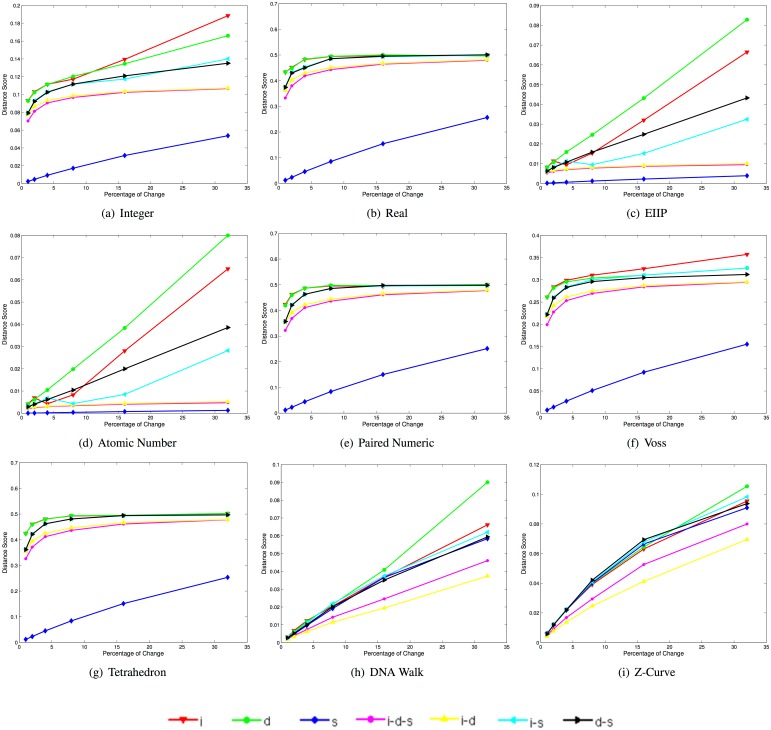
Mean Normalized squared euclidean distance scores for 400 synthetic sequences in each of the 42 datasets when using each of the selected DNR (i: insertion, d: deletion, s: substitution, i-d-s: insertion-deletion-substitution, i-d: insertion-deletion, i-s: insertion-substitution, d-s: deletion-substitution).

Fig 4 depicts plots of the mean normalized squared Euclidean distance in the same synthetic data set. Note that the integer, EIIP and atomic number representations present a similar behavior to that observed when using the Euclidean distance. The real, paired numeric and tetrahedron representations present very small differences for deletions, deletions-substitutions, and insertion-substitutions after approximately 15% of changes, which may make it unreliable for differentiating among such types of changes. The Voss representation appears to preserve the same structure as with the Euclidean distance, but with more noticeable differences in the distances between the deletion-substitutions, insertion-deletions and insertion-deletion-substitutions. Moreover, it is noteworthy that the cumulative DNRs are more sensitive to deletions, compared to the large sensitivity to insertions when using the Euclidean distance.

Table 3 lists the the angle (in degrees) of the rate of change in the mean normalized squared Euclidean distance scores for the type of change corresponding to insertions-deletions-substitutions for five ranges of the percentage of change. Note that the angle of the rate of change is relatively small for all DNRs compared to the angle of the rate of change observed when using the unbounded Euclidean distance (Table 2). This is explained by the normalization step, which bounds the maximum possible score to the value of one, and therefore, has the effect of “compressing” the relative difference scores.

**Table 3:** Angle (in degrees) of the rate of change in the mean normalized squared Euclidean distance scores for the type of change corresponding to insertions-deletions-substitutions, for five ranges of the percentage of changes (2-1, 4-2, 8-4, 16-8, 32-16). *A* = ×10^−2^, *B* = ×10^−3^.

	Angle of the Rate of Change					Score by Percentage of Change					
DNR	2-1	4-2	8-4	16-8	32-16	1	2	4	8	16	32
Integer	0.6	0.3	8.8^*A*^	4.1^*A*^	1.5^*A*^	0.07028	0.08121	0.09058	0.09671	0.10250	0.10663
Real	2.7	1.1	0.3	0.2	5.4^*A*^	0.33235	0.37968	0.41860	0.44303	0.46433	0.47933
EIIP	5.0^*A*^	2.5^*A*^	1.1^*A*^	6.4^*B*^	3.0^*B*^	0.00530	0.00618	0.00707	0.00780	0.00870	0.00954
Atomic Number	2.5^*A*^	1.4^*A*^	6.1^*B*^	4.7^*B*^	2.3^*B*^	0.00208	0.00251	0.00300	0.00342	0.00408	0.00473
Paired Numeric	2.7	1.2	0.4	0.2	5.9^*A*^	0.32230	0.36861	0.41081	0.43616	0.46088	0.47739
DNA Walk	0.1	9.8^*A*^	9.9^*A*^	7.4^*A*^	7.7^*A*^	0.00210	0.00390	0.00734	0.01422	0.02453	0.04605
Voss	1.6	0.7	0.2	0.1	3.8^*A*^	0.19917	0.22771	0.25325	0.26935	0.28420	0.29469
Tetrahedron	2.6	1.2	0.3	0.2	5.9^*A*^	0.32567	0.37133	0.41189	0.43632	0.46108	0.47756
Z-Curve	0.2	0.2	0.2	0.2	9.8^*A*^	0.00506	0.00917	0.01684	0.02940	0.05263	0.07996

**Fig 5:**
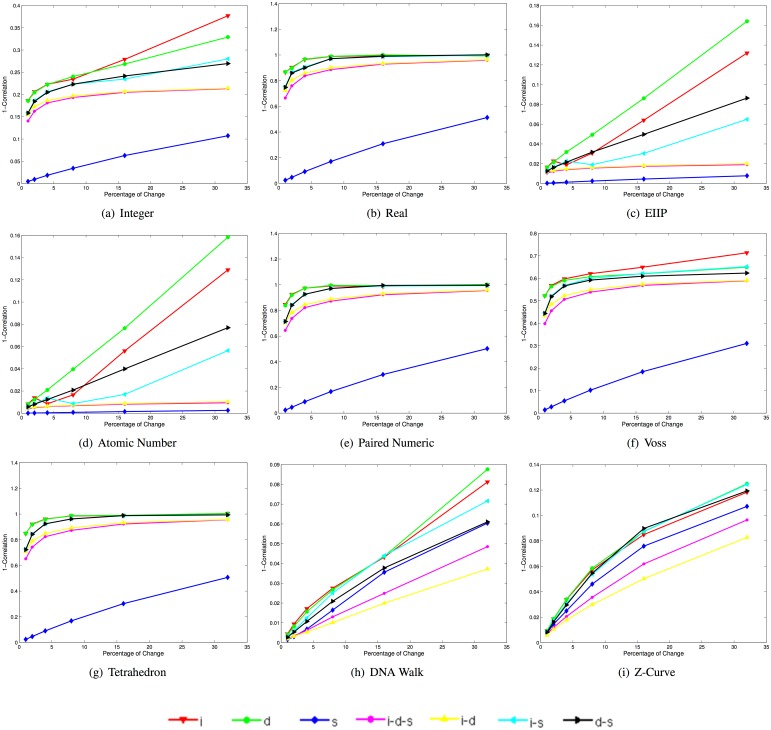
Mean Manhattan distance scores for 400 synthetic sequences in each one of the 42 datasets when using each of the selected DNR (i: insertion, d: deletion, s: substitution, i-d-s: insertion-deletion-substitution, i-d: insertion-deletion, i-s: insertion-substitution, d-s: deletion-substitution).

Fig 5 depicts the results corresponding to the use of the Manhattan distance in the synthetic DNA signal data set. Note that for all DNRs the substitutions present the highest similarity with respect to the original sequence, while the insertions represent the largest differences. Moreover, the order of the curves in the plots indicate that this distance may be more robust with respect to the DNRs employed.

Table 4 lists the the angle (in degrees) of the rate of change in the Manhattan distance scores for the type of change corresponding to insertions-deletions-substitutions for five ranges of the percentage of change. Note that the angles remain large for all the DNRs with the exception of the EEIP, compared to the Euclidean distance, which means that the difference score will continue to increase as the percentage of change increases.

**Table 4:** Angle (in degrees) of the rate of change in the mean Manhattan distance scores for the type of change corresponding to insertions-deletions-substitutions, for five ranges of the percentage of changes (2-1, 4-2, 8-4, 16-8, 32-16).

	Angle of the Rate of Change					Score by Percentage of Change					
DNR	2-1	4-2	8-4	16-8	32-16	1	2	4	8	16	32
Integer	90.0	89.9	89.7	89.3	88.0	14545.7	15922.5	17065.4	17796.3	18440.1	18891.6
Real	90.0	89.9	89.6	89.2	87.5	14127.3	15379.3	16405.9	17043.9	17589.7	17949.1
EIIP	88.1	85.7	77.5	66.1	40.2	302.3	331.9	358.3	376.4	394.5	408.0
Atomic Number	90.0	90.0	90.0	89.9	89.9	109659.5	121566.4	132437.8	139022.9	147648.2	154013.4
Paired Numeric	89.9	89.9	89.6	89.1	87.3	12759.7	13901.9	14878.3	15468.6	15958.7	16297.6
DNA Walk	90.0	90.0	89.9	89.9	89.9	19099.4	23006.4	27651.7	32232.5	37815.1	45259.6
Voss	89.9	89.7	89.1	88.0	84.2	5532.5	6023.3	6443.3	6704.3	6939.2	7097.1
Tetrahedron	89.9	89.8	89.3	88.5	85.5	7375.7	8020.8	8575.5	8902.9	9217.5	9421.4
Z-Curve	90.0	90.0	90.0	89.9	89.9	18633.5	22596.3	27166.0	31891.5	38082.2	44335.7

**Fig 6:**
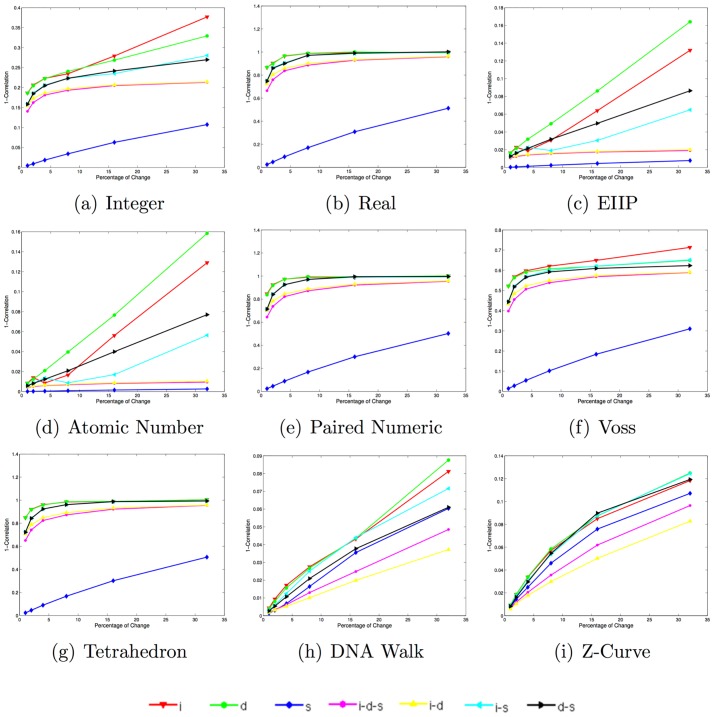
Mean 1-Correlation scores for 400 synthetic sequences in each one of the 42 datasets when using each of the selected DNRs. **i-d:** insertion-deletion, **i-s:** insertion-substitution, **d-s:** deletion-substitution). Note that the range for each box is not between [0, 1], instead they vary in order to present a better visualization.

Fig 6 depicts the mean complementary correlation coefficient scores (i.e., 1-Correlation) for the same data when using each one of the selected DNRs.

Note that the magnitude of the similarity scores, in particular the EIIP, the atomic number, and the cumulative representations, present high correlation scores even when large changes occur (e.g., a correlation of approximately 0.94 for changes of 32% with the baseline sequence in the curve corresponding to deletions). Note that for all the non-cumulative DNRs, the highest mean correlation score is obtained by the type of change corresponding to substitutions.

The mean correlation coefficient corresponding to the other types of changes behaves similarly for the real, paired Numeric, and Tetrahedron representations, with the curve corresponding to substitutions far above the other curves, and an apparent convergence of these curves as the percentage of changes increases. The Voss representation behaves similarly, with the difference that the mean correlation coefficient scores are higher for all the curves, and a better separation of the other curves as the percentage of changes increases, as well as its distinct behavior for insertions, which is most similar to the one for the integer representation.

The integer representation also behaves similarly, with the difference that the other curves score higher than the Voss representation curves. The EIIP and Atomic number curves behave like each other, with minor differences in the mean correlation coefficients. Finally, the DNA-walk and Z-curve representations present a quasi-linear decrease in their mean correlation scores with respect to increasing percentages of changes.

For these DNRs, the highest score is obtained by the insertion-deletion curve. Unlike the other DNRs, the i-d-s and i-d curves are better separated. Note how these results are consistent with those obtained with the other distances, with the main difference that the correlation coefficient is always in the range of [−1, 1], while the Euclidean and Manhattan distances ranges within [0, ∞) and the normalized Euclidean distance in the range of [0, 1].

Table 5 lists the angle (in degrees) of the rate of change in the mean correlation coefficient scores for the type of change corresponding to insertions-deletions-substitutions for five ranges of the percentage of change.

**Table 5:** Angle (in degrees) of the rate of change in the mean correlation coefficient scores for the type of change corresponding to insertions-deletions-substitutions, for five ranges of the percentage of changes (2-1, 4-2, 8-4, 16-8, 32-16). *A* = ×10^−2^, *B* = ×10^−3^.

	Angle of the Rate of Change					Score by Percentage of Change					
DNR	2-1	4-2	8-4	16-8	32-16	1	2	4	8	16	32
Integer	1.3	0.5	0.2	8.3^*A*^	3.0^*A*^	0.14053	0.16240	0.18112	0.19337	0.20493	0.21317
Real	5.4	2.2	0.7	0.3	0.1	0.66464	0.75931	0.83715	0.88604	0.92864	0.95865
EIIP	0.1	5.1^*A*^	2.1^*A*^	1.3^*A*^	6.0^*B*^	0.01060	0.01236	0.01413	0.01560	0.01739	0.01907
Atomic Number	5.0^*A*^	2.8^*A*^	1.2^*A*^	9.5^*B*^	4.7^*B*^	0.00416	0.00503	0.00599	0.00684	0.00816	0.00946
Paired Numeric	5.3	2.4	0.7	0.4	0.1	0.64453	0.73715	0.82156	0.87229	0.92173	0.95477
DNA Walk	8.9^*A*^	8.0^*A*^	9.7^*A*^	8.5^*A*^	8.5^*A*^	0.00186	0.00341	0.00621	0.01300	0.02488	0.04852
Voss	3.3	1.5	0.5	0.2	7.5^*A*^	0.39826	0.45532	0.50639	0.53857	0.56826	0.58921
Tetrahedron	5.2	2.3	0.7	0.4	0.1	0.65123	0.74256	0.82370	0.87258	0.92212	0.95510
Z-Curve	0.3	0.2	0.2	0.2	0.1	0.00691	0.01187	0.02045	0.03559	0.06183	0.09648

Similar to the case of the normalized squared Euclidean distance, the angles are subtle for almost all DNRs. Note that for the Atomic number, EEIP, and DNA-Walk representations, the angles are near zero for every range of percentage of change. Therefore, the similarity between two sequences may be impossible to estimate using these DNRs with this metric. The integer representation method may be more sensitive to differences between two signals, while the real, paired numeric, and cumulative representations may be a better option for estimating the correlation between two sequences.

**Fig 7:**
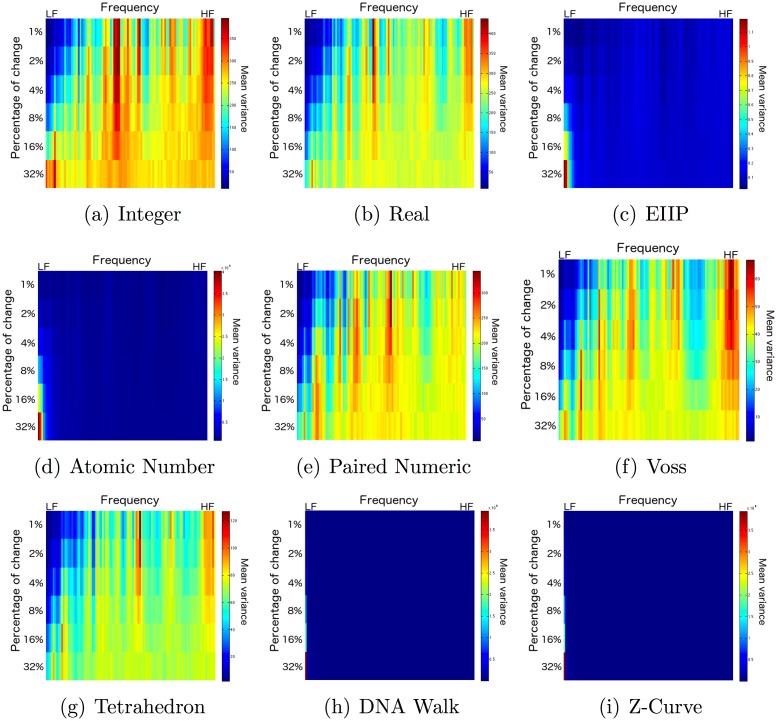
Mean variance of the frequency components according to the percentage of change for the selected DNRs using a color palette where red and blue represent high and low variances, respectively. HF stands for high and and LF for low frequencies.

Fig 7 depicts the mean variance of the frequency components according to the percentage of change for the selected DNRs using a color palette where red and blue represent high and low variances, respectively. Note that the tetrahedron representation concentrates the variability around the higher frequencies as well as the frequency corresponding to approximately 1/5 of the maximum frequency for percentages of change around 8%, and more homogeneous spread of variability for higher percentages of change. The integer, real, and Voss representations have a significant variability in the high-frequency components, and in some of the low- and mid- frequency components.

The paired numeric representation concentrates the variability in the mid-frequency components. The EIIP, atomic number, and cumulative representations concentrate an extremely high variability in the low frequencies for percentages of change larger than 8%, which depresses the variability in the other frequency components among the remaining percentages of change. This explains the high correlation scores for these DNRs, since almost the entire power spectrum may seem similar in comparison to the largest possible value differences for the low frequency components.

Table 6 lists the scores for the comparison of the synthetic baseline sequence with its corresponding complementary sequence, and the reverse complementary sequence. Note that the real and paired numeric representations obtain scores that indicate the identity of the power spectra of the complementary and reverse complementary sequences for all metrics. This can be explained because these DNRs consider the complementarity property of the DNA strands for the numeric mapping and, therefore, generate the same patterns in the signals.

**Table 6:** Complementary sequence scores for each DNR. EC stands for Euclidean distance, CC for correlation coefficient, NE for normalized Euclidean distance, and MD for Manhattan distance.

	Complementary				R. complementary			
DNR	ED	NE	MD	CC	ED	NE	MD	CC
Integer	636.92	0.075	1.63×10^4^	0.84	636.92	0.075	1.63×10^4^	0.84
Real	0	0	0	1	0	0	0	1
EIIP	15.68	0.008	391.46	0.98	15.68	0.008	391.46	0.98
Atomic number	4.69×10^3^	0.002	1.02×10^5^	0.99	4.69×10^3^	0.002	1.02×10^5^	0.99
Paired numeric	0	0	0	1	0	0	0	1
Voss	277.12	0.289	7.07×10^3^	0.42	277.12	0.289	7.07×10^3^	0.42
Tetrahedron	245.93	0.316	6.25×10^3^	0.36	245.93	0.316	6.25×10^3^	0.36
Z-Curve	0	0	0	1	8.98×10^3^	0.081	3.62×10^4^	0.8925
DNA Walk	0	0	0	1	1.42×10^4^	0.113	4.74×10^4^	0.8837

This behavior may be an advantage in some cases of analysis where it is desirable to account for the structural complementarity of the DNA (for example, for determining the similarity between two DNA sequences **A** and **B** without the need to determine which of the two strains of **A** or **B** needs to be employed). However, this may be a disadvantage in cases where a detailed analysis of the differences between two DNA sequences is required.

Note that the Z-curve and DNA Walk representations also provide scores that indicate identical power spectra compared to the complementary sequence. However, in the case of the reverse complementary sequences, these two scores indicate a large difference between their frequencies. This property can be explained by the cumulative characteristic of these DNRs, which generates different DNA signals when taking the reverse direction. This response could represent an important criticism of these DNRs, since in their formulation the authors justify the mapping values employed arguing that they consider the DNA complementarity property, while this does not apply for computing similarities. Integer, EIIP, Atomic Number, Voss and Tetrahedron present the same scores for the complementary sequence and the reverse complementary sequence, respectively. The latter is due to the symmetry property of the frequency spectrum (i.e., the frequency spectrum of a numeric sequence is the same even if this numeric sequence is sorted in reverse order).

Voss does not present such behavior because of the procedure used to transform a multidimensional signal to a single-dimensional signal in which, for each dimension, the power spectrum is computed and then concatenated one after the other.

### Biological data results

**Fig 8:**
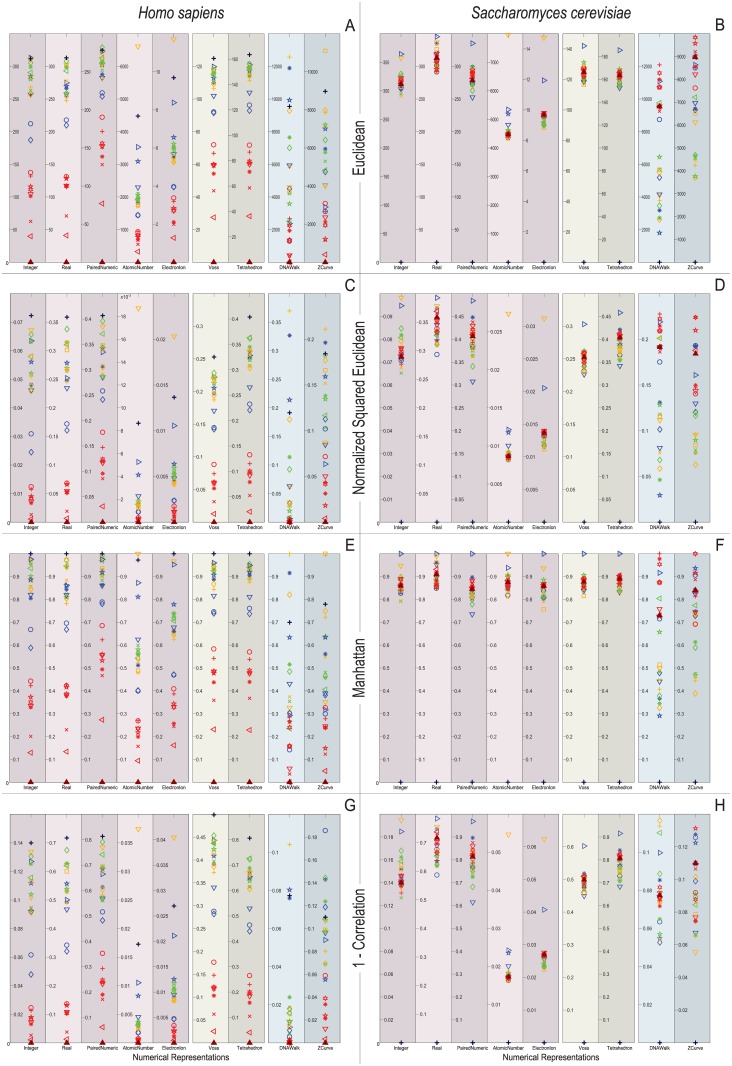
Biological experiment results for the similarity computation of the selected gene RP-S18 sequences with respect to *H. sapiens* (left column) and *S. Cerevisiae* (right column) when using the four selected similarity metrics.

Fig 8 depict the distribution of the similarity scores of all the selected species of the gene RP-S18 with respect to *H. sapiens* (left column) and *S. Cerevisiae* (right column) when using the four selected similarity metrics. Note that all the non-cumulative DNRs were successful in clustering all mammals with a large similarity score when compared to *H. sapiens*. Also, note that the *Macaca mulatta* and *Pan Troglodytes* were the closest species to *H. sapiens* as was to be expected.

When using the Euclidean distance, only the Real, Voss, and Tetrahedron representations successfully assign a lower similarity score to the *S. Cerevisiae* than to all other species (i.e., the black cross marked on top of all other markers). However, when using the normalized squared Euclidean distance and the correlation coefficient, the integer, real, Paired Numeric, Voss, and tetrahedron representations depict the black cross above every other marker. When using the Manhattan distance, the EEIP also depict the *S. Cerevisiae* as the most unrelated specimen. Note that for the non-cumulative DNRs all species tend to cluster together with low similarity scores when compared to *S. Cerevisiae*. DNA walk and Z-curve do not show this clustering and present a more uniform distribution of the similarity scores for all metrics. When using the correlation coefficient, a similar behavior can be observed, with the main difference being in the Atomic number and EEIP representations where the species are grouped with a high similarity score when compared to *S. Cerevisiae* (i.e., around 98% to 99% correlation). This implies that all species are very similar to *S. Cerevisiae*, which is incorrect. Similarly, the cumulative representations yield high similarity scores with respect to this species.

**Fig 9:**
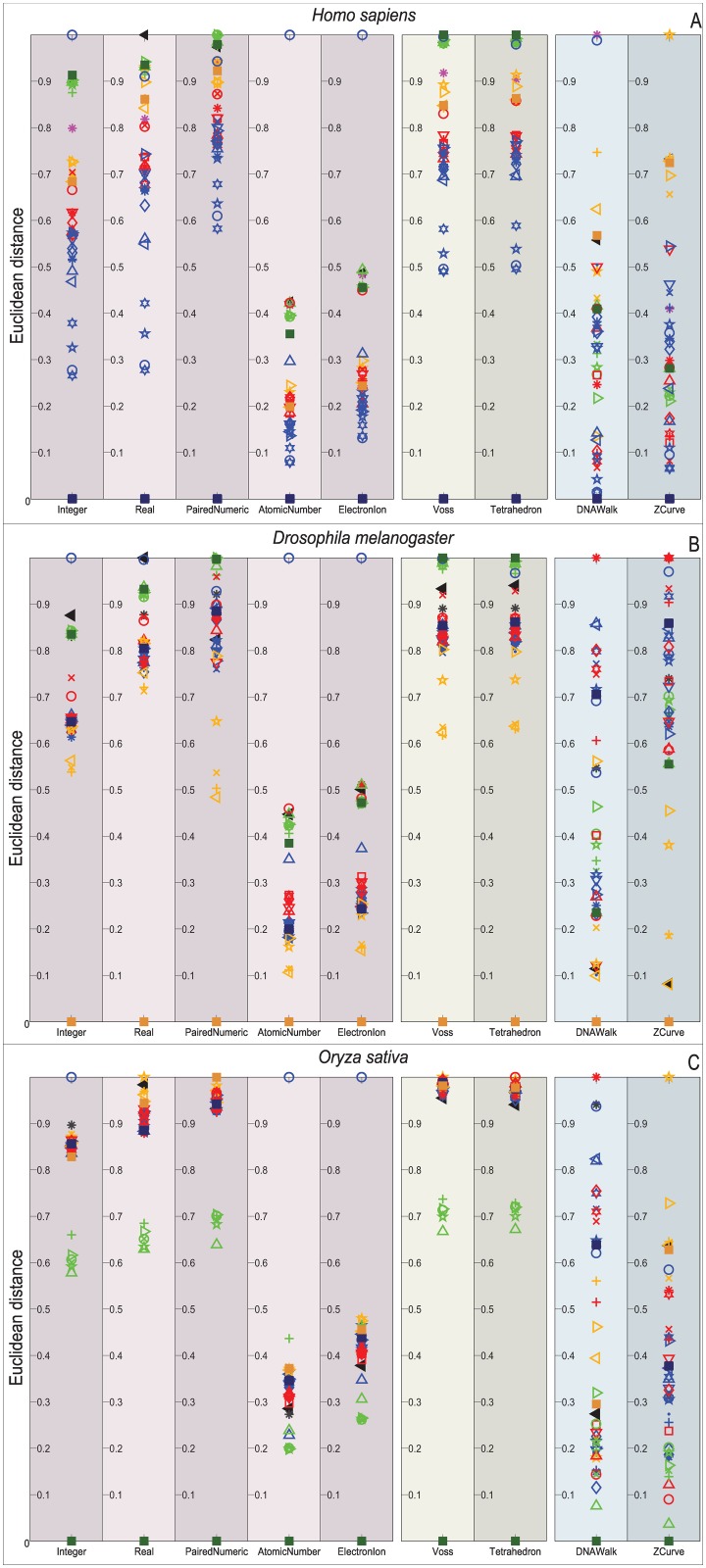
Biological experiment results for the similarity computation of the selected gene COX1 sequences with respect to *H. sapiens* (top), *Drosophila melanogaster* (middle), and *Oryza sativa* (bottom) when using the Euclidean distance as the similarity metric.

**Fig 10:**
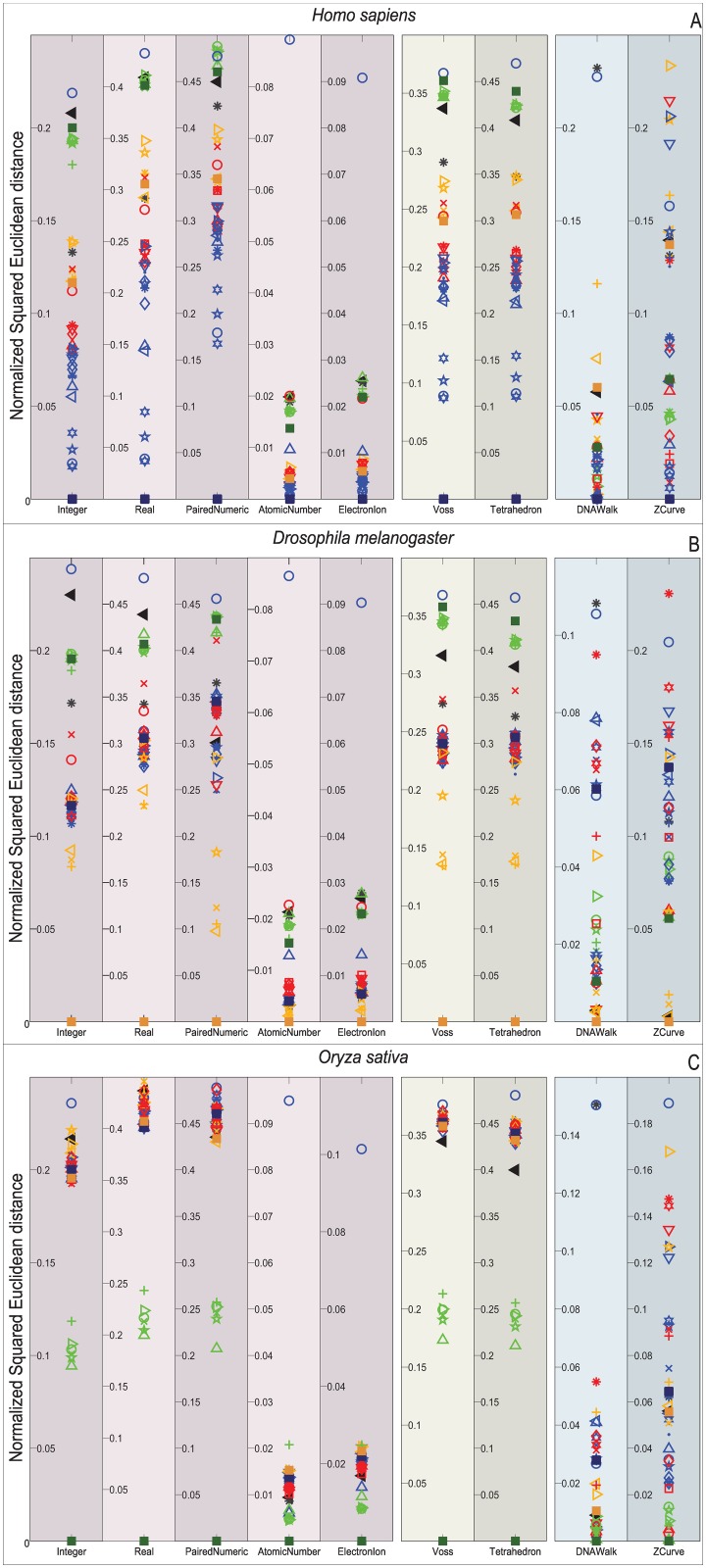
Biological experiment results for the similarity computation of the selected gene COX1 sequences with respect to *H. sapiens* (top), *Drosophila melanogaster* (middle), and *Oryza sativa* (bottom) when using the normalized squared Euclidean distance as the similarity metric.

**Fig 11:**
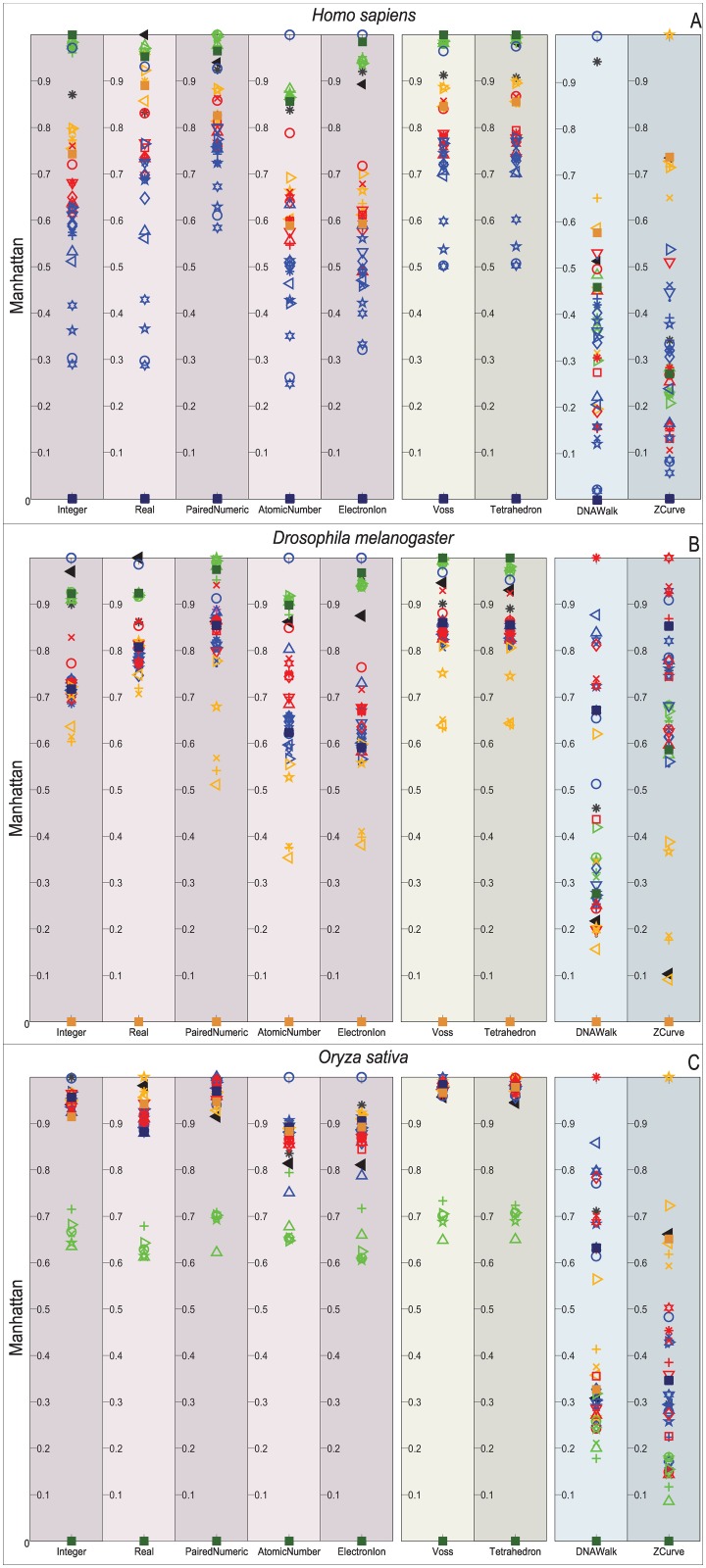
Biological experiment results for the similarity computation of the selected gene COX1 sequences with respect to *H. sapiens* (top), *Drosophila melanogaster* (middle), and *Oryza sativa* (bottom) when using the Manhattan distance as the similarity metric.

**Fig 12:**
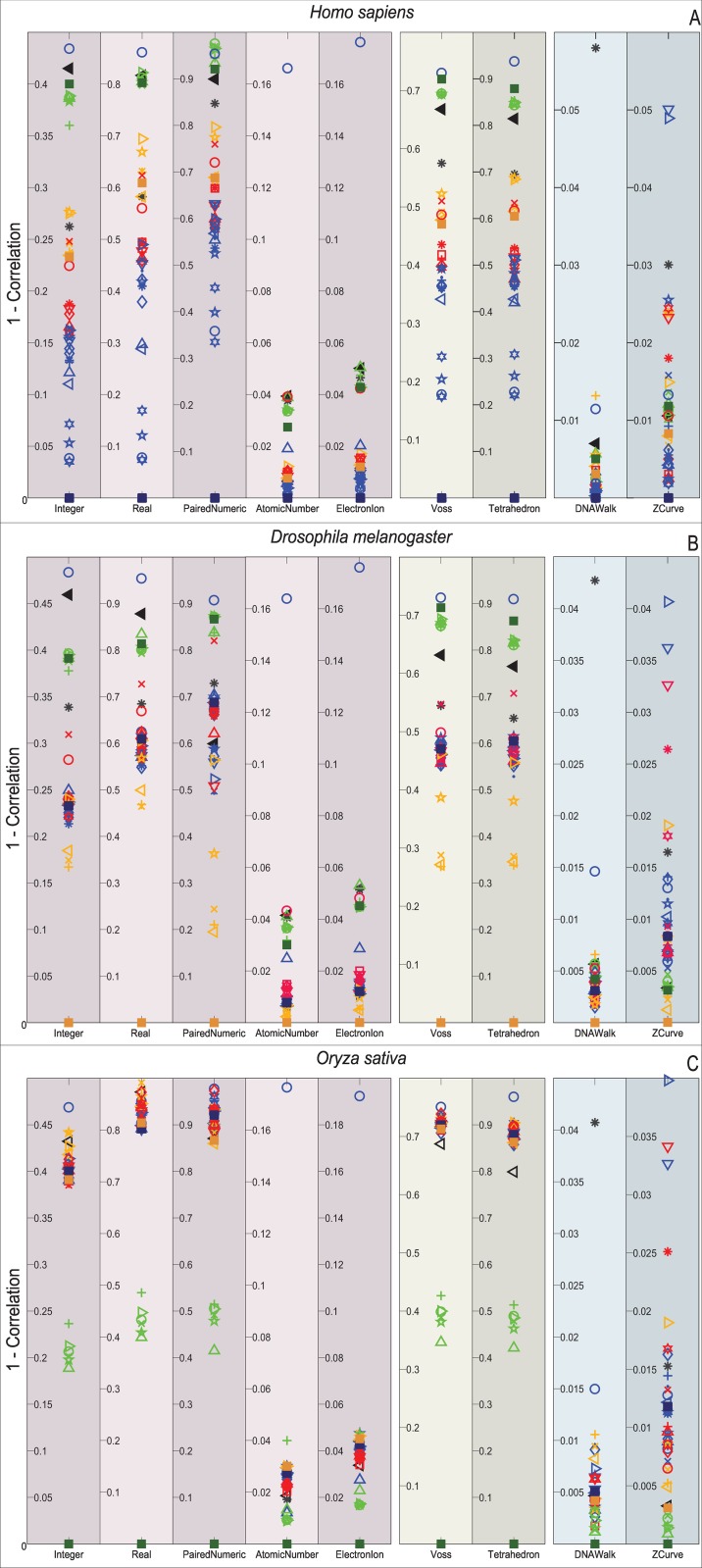
Biological experiment results for the similarity computation of the selected gene COX1 sequences with respect to *H. sapiens* (top), *Drosophila melanogaster* (middle), and *Oryza sativa* (bottom) when using the correlation coefficient as the similarity metric.

Figs 9–12 depict the distribution of the similarity scores of all the selected species of the gene COX1 with respect to *H. sapiens*, (B) *Drosophila melanogaster*, and (C) *Oryza sativa* when using the Euclidean distance, squared Euclidean distance, Manhattan distance, and correlation coefficient as the similarity metrics, respectively.

Note that overall, the distance measurements remain similar for the single and multidimensional representations. However, this is not the case for the cumulative DNRs that smear all the species without any chance to resolve even at the Phylum level. In contrast, the Atomic Number and EEIP representations present an erratic clustering of the taxa.

Note that the main difference of all the explored distance metrics is the scale at which they differentiate the organisms, following from the lowest-to-highest: Euclidean < Correlation < Norm L2 < Manhattan. At first glance, the Manhattan distance may seem to disperse adequately through the relevant order layers, but when reviewed for all the comparisons it becomes clear that this measurement is quickly saturated and renders maximum distances to groups that the COX1 gene may still differentiate at the phylum level. Likewise, the Norm L2 distance can barely differentiate between the Phyla before reaching saturation points.

An interesting result is that *Bos mutus* is consistently the farthest specimen on almost every comparison, independently of the DNR and distance measurement employed. When performing a more detailed examination of its respective KEGG entry (bom:102267288) it showed that even when it is a COX1 gene, in the RefSeq is registered as cytochrome c oxidase subunit 1-like. This means that, a distant homologous gene was introduced and it acted as the external group since it showed greater distance than *Saccharomyces cerevisiae*. This shows that the methodology presented in this work is capable of discriminating between close orthology and more distant homologies.

## Discussion

The proposed DNRs may be grouped into two categories, according to the values to be assigned to each nucleotide [18]: fixed value-based mapping methods characterized by employing arbitrary numeric values for each DNA letter, and biological-based mappings characterized by their use of numerical values that are somehow justified by some biochemical or biophysical properties of the DNA molecules.

We believe that the robustness of the fixed value-based mapping methods such as the integer and real representations is questionable since they do not consider any biological property. Moreover, it is evident that the use of different values generates different results. If we look at EIIP and atomic number representations as fixed value mapping methods since they employ characteristics that may not directly affect the biological properties or the dynamics associated with the DNA molecules, we can verify that the use of arbitrary values and intervals lead to different results. In that respect, biological-based mappings such as the Voss and tetrahedron representations which consider the properties of the DNA molecules and their interactions may represent a better choice.

In the research presented in this paper, we performed experiments employing synthetic DNA sequences that were generated and altered with different types of change in a cumulative manner, using a uniform probability distribution for the selection of each type of nucleotide. This procedure may not be valid for modeling real biological DNA sequences, since the relative proportions of bases in DNA are not even [41]. However, given that the numeric values assigned to each nucleotide are different among the selected DNRs, the uniform probability distribution employed seems to be appropriate to avoid a possible bias in the results due to a high frequency of appearance of a certain numeric value.

It is interesting that the EIIP and atomic number representations behave similarly to each other, and unlike the rest of the single-dimensional DNRs (Figs 3–6). We believe this is because of the cost of change of a nucleotide, in a given sequence, to a different one. Such a cost is determined by the arithmetical difference in the value of the two different nucleotides to be interchanged (the larger the difference, the greater the cost). In the case of the integer, real, and paired numeric representations, the costs are relatively lower, in comparison with the cost when using the EIIP and atomic number representations. In these latter DNRs, large differences between sequences will tend to generate disproportionately lower frequencies, as can be verified in Fig 7.

It is thus evident that the cumulative representations obtained the worst results with respect to our hypothesis. In particular, we believe that these types of representations are not suitable for FFT-based GSP methods, because of their lack of stationarity, which is a desideratum when using digital signal processing methods [42]. Moreover, the cumulative representations tend to generate disproportionately greater lower frequencies, similarly to the EIIP and atomic number representations (Fig 7).

In this sense, the multidimensional representations may be considered as more appropriate choices, since their structure makes it possible to have equal costs for the replacement of any two nucleotide types. From the results obtained using biological data, we verified that, indeed, the multi-dimensional representations are more accurate with respect to what was expected as a result of the biological experiments. The paired numeric and real representations also seem to be adequate for GSP, since they consider the structural characteristics of the DNA molecule (i.e., complementarity property). This can be verified as well in the biological results (Figs 8–12).

In fact, we can verify that all the non-cumulative selected DNRs are sub-spaces of the space generated by the Voss representation. For example, the integer, real, EEIP, atomic number, and paired numeric representations can be derived from the Voss representation by multiplying each Voss indicator sequence by the values assigned to each nucleotide type on each of the DNRs, and then performing a sum over the four dimensions.

From the results obtained in this research, we believe that an adequate DNR could consist of a multidimensional mapping that employs different values corresponding to the biological properties of the DNA molecules in each dimension. Moreover, we believe that the notion of neighboring nucleotides must be considered. In this sense, the use of the *k*-tuples approach could be useful when defining a new DNR.

An application of the presented approach is the assessment of the similarity among sets of DNA sequences without the need of performing alignment over the DNA characters. This will allow performing faster comparisons among large databases, especially if the sequences are stores in DNA signal form with their corresponding power spectra. In fact, thanks to the increase of algorithms and computational methods based on the use of Graphical Processing Units (GPU), we believe that it is very likely that most of the GSP methods will be based on these technologies. Our future work includes the implementation of our methods using GPU and, the evaluation and development of additional DNRs and methods for DNA analysis based on GSP techniques.

## Conclusion

We have presented an experimental study on the characteristics of nine DNRs belonging to three categories. Our results indicate that the multidimensional DNRs such as the Voss and tetrahedron representations are more appropriate for the computation of the similarity between DNA signals than are the other DNRs.

## Supporting information

S1 File MatlabCode.zip. Matlab code and scripts for running the experiments we describe in this work.
(ZIP)

S2 File libraries.zip. Matlab functions needed for running the experiments we describe in this work.
(ZIP)

S3 File datasets.zip. The datasets employed in this work.
(ZIP)

S4 File README.txt. Instructions of how to use the code and run the experiments.
(TXT)
